# Editorial: Bacteriophages to Fight Food-Borne Pathogens/Phages Struggling for Food Safety

**DOI:** 10.3389/fmicb.2021.741387

**Published:** 2021-09-23

**Authors:** Claudia Picozzi, Pilar Garcia, Martha Vives

**Affiliations:** ^1^Dipartimento di Scienze per gli Alimenti, la Nutrizione e l'Ambiente (DeFENS), Università degli Studi di Milano, Milano, Italy; ^2^Departamento de Tecnología y Biotecnología de Productos Lácteos, Instituto de Productos Lácteos de Asturias, Instituto de Productos Lácteos de Asturias, Consejo Superior de Investigaciones Científicas (IPLA-CSIC), Villaviciosa, Spain; ^3^Department of Biological Sciences, Universidad de los Andes, Bogotá, Colombia

**Keywords:** bacteriophages, food safety, phage therapy, biocontrol, phage cocktails

In recent years, the application of phage therapy for the control of pathogenic bacteria has been experiencing a new renaissance driven by the emergence of multidrug-resistant bacteria (Gordillo Altamirano and Barr, 2019). Bacteriophages are natural predators of bacteria, presenting several benefits, the main ones related to their high specificity and their harmlessness to humans, animals, and plants. The interactions between phages and their hosts play an important role in the evolutionary ecology mechanisms of bacterial resistance and phage infectivity (“antagonistic coevolution”). Some failures in the application of phage therapy are indeed due to bacterial mutations that led to resistance (Buckling and Rainey, 2002).

To be considered as excellent candidates as biocontrol agents in foods, phages should be strictly lytic and possibly have a broad host range. To overcome this limitation, formulations with more than one phage (phage cocktails) can be used. But how many and which isolates should compose these cocktails? The optimum formulation should provide a compromise between achieving a high reduction on the bacterial load and minimizing the side effects (cost of production, dysbiosis, risk of horizontal transfer of toxins, antibiotic resistance, or virulence genes) of an increasing cocktail complexity. Molina et al. tackled these challenging questions proposing a new pipeline for designing phage cocktails. Their analysis started with the building of a phage-bacteria infection network (PBIN) from host range matrices using two different algorithms, a genetic and a heuristic one. The first one led to the lower temperature, i.e., increased nestedness, simplifying the identification of phages with the broadest host range. They performed a meta-analysis of 35 host range matrices and proposed a new metric (Φ) for estimating phage cocktail size. At the end they applied an agglomerative hierarchical clustering on their data, developing a cocktail that was able to reduce bacterial counts of cheese-isolated *Escherichia coli* of five orders of magnitude. To further validate these results, Molina et al. submitted another work where they characterized coliphages isolated from ewe feces and *E. coli strains* isolated from goat and sheep raw milk cheeses. Several phages showed a broad host range, making them good candidates for *E. coli* biocontrol. However, they highlighted that phage virulence decreased as bacterial susceptibility range increased, until reaching a plateau, revealing a local gene-for-gene coevolution between hosts and phages that explained the ability of the phages they isolated to propagate on host strains from different niches. They concluded that, when devising long-term and short-term biocontrol strategies, different phage cocktail formulations might be required. Their results affirm the importance of knowing the specific interactions between phages and bacteria to develop successful phage applications.

The formulation of a cocktail is also the basis of the work of Mangieri et al. which shows the relevance of this particular step in any phage therapy endeavor. The aim of the authors was to formulate a cocktail able to reduce the counts of pathogenic *E. coli* (Shiga toxin-producing *E. coli*-STEC) in food. They analyzed 20 different bacteriophages isolated from feces, sewage, and bedding material for their lytic ability toward a population of STEC strains belonging to different serogroups and with different antibiotic-resistance profiles. Three of these phages were selected, also taking into account their RAPD (Random Amplification of Polymorphic DNA) profile and the absence of antibiotic-resistance genes and virulence-encoding genes. This cocktail was then tested directly on a food matrix (cucumber) at different temperatures resulting in a 2-log reduction of *E. coli* cells after 24 h both at 4 and 25°C, suggesting possible application in biocontrol of different STEC serogroup on raw products and RTE foods. Considering that foodborne diseases were estimated to cause 600 million illnesses resulting in 420,000 deaths (Havelaar et al., 2015), the possibility to use bacteriophages and their derivatives to prevent or kill food pathogens is extremely crucial. This work shows the potential of using phages in fresh vegetables and opens new possibilities for upcoming studies on its applications.

Another important benefit of bacteriophages is that they will not cause any problem in consumers nor affect the taste, color, smell, and texture of the food products. Starting from this assumption, Ahmadi et al. investigated the effectiveness of using bacteriophages added as an ingredient in a cooked meat product to control the growth of *Listeria monocytogenes*. They elaborated different scenarios for bacteriophage application and pathogen contamination where phage and sensitive strain were introduced inside or on the surface of the cooked meat stored at 4°C. Only when phage was applied directly on top of *L. monocytogenes* inoculated on the surface of cooked meat was there a reduction in cell number below the detection limit. As concerning other applications where phage and bacteria were mixed and inoculated in meat or when only the bacteriophage was inoculated in meat and *L. monocytogenes* spread on the surfaces, or *vice versa*, they were not able to obtain any reduction in cell numbers. The main reason is probably the immobilization of phages and bacteria inoculated in a solid food matrix that limits bacteriophage-host interactions. Therefore, more studies are needed to increase the access of phages to their hosts in complex matrixes, and integration of fields such as food technology, nanomaterials, active compound-controlled delivery, and food compatible bio-emulsifiers.

Overall, bacteriophages undoubtedly have a great potential in a food safety context. Since more research is necessary to achieve a suitable cocktail or phage-derived product to cover the current industrial sector needs, this emerging and exciting area is an open field for interdisciplinary research and innovations.

## Author Contributions

All authors listed have made a substantial, direct and intellectual contribution to the work, and approved it for publication.

## Conflict of Interest

The authors declare that the research was conducted in the absence of any commercial or financial relationships that could be construed as a potential conflict of interest.

## Publisher's Note

All claims expressed in this article are solely those of the authors and do not necessarily represent those of their affiliated organizations, or those of the publisher, the editors and the reviewers. Any product that may be evaluated in this article, or claim that may be made by its manufacturer, is not guaranteed or endorsed by the publisher.
